# Blue-Light Therapy for Seasonal and Non-Seasonal Depression: A Systematic Review and Meta-Analysis of Randomized Controlled Trials

**DOI:** 10.1177/07067437221097903

**Published:** 2022-05-06

**Authors:** André Do, Victor W. Li, Samantha Huang, Erin E. Michalak, Edwin M. Tam, Trisha Chakrabarty, Lakshmi N. Yatham, Raymond W. Lam

**Affiliations:** 1 Department of Psychiatry, 8166 University of British Columbia, Vancouver, Canada

**Keywords:** blue-light therapy, seasonal depression, non-seasonal depression, seasonal affective disorder, major depressive disorder, wavelength

## Abstract

**Objectives:**

To determine the efficacy and safety of blue-light therapy in seasonal and non-seasonal major depressive disorder (MDD), by comparison to active and inactive control conditions.

**Methods:**

We searched Web of Science, EMBASE, Medline, PsycInfo, and Clinicaltrials.gov through January 17, 2022, for randomized controlled trials (RCTs) using search terms for blue/blue-enhanced, light therapy, and depression/seasonal affective disorder. Two independent reviewers extracted data. The primary outcome was the difference in endpoint scores on the Structured Interview Guide for the Hamilton Depression Rating Scale - Seasonal Affective Disorder (SIGH-SAD) or the Structured Interview Guide for the Hamilton Depression Rating Scale with Atypical Depression Supplement (SIGH-ADS) between blue light and comparison conditions. Secondary outcomes were response (≥ 50% improvement from baseline to endpoint on a depression scale) and remission rates (endpoint score in the remission range).

**Results:**

Of 582 articles retrieved, we included nine RCTs (*n* = 347 participants) assessing blue-light therapy. Seven studies had participants with seasonal MDD and two studies included participants with non-seasonal MDD. Four studies compared blue light to an inactive light condition (efficacy studies), and five studies compared it to an active condition (comparison studies). For the primary outcome, a meta-analysis with random-effects models found no evidence for the efficacy of blue-light conditions compared to inactive conditions (mean difference [MD] = 2.43; 95% confidence interval [CI], −1.28 to 6.14, *P* = 0.20); however, blue-light also showed no differences compared to active conditions (MD = −0.11; 95% CI, −2.38 to 2.16, *P* = 0.93). There were no significant differences in response and remission rates between blue-light conditions and inactive or active light conditions. Blue-light therapy was overall well-tolerated.

**Conclusions:**

The efficacy of blue-light therapy in the treatment of seasonal and non-seasonal MDD remains unproven. Future trials should be of longer duration, include larger sample sizes, and attempt to better standardize the parameters of light therapy.

## Introduction

Light therapy consists of daily exposure to bright fluorescent light that is typically delivered at home via a light device such as a light box. Light therapy has been extensively studied in seasonal affective disorder (SAD), but increasing evidence suggests that it is also effective in non-seasonal major depressive disorder (MDD) and bipolar depression.^1,2^ Systematic reviews and meta-analyses have found a moderate effect size for light therapy compared to inactive controls, although the significant heterogeneity and the small sample sizes of the included studies were highlighted as limitations.^3–5^ The standard protocol for light therapy for depression uses white light at an intensity of 10,000 lux for 30 min per day during the early morning for up to 6 weeks.^6^ Lux is a measure of illumination that varies with the distance to the light source. For comparison, indoor social lighting is rated as less than 100 lux, bright office lighting at 500 lux, outdoors on a cloudy day at 5,000 lux, and outdoors on a sunny day at 50,000 lux or higher.^7^ Light therapy is generally well-tolerated with few or mild side effects.^8^

Although the precise mechanism of action of light therapy remains unclear, it is hypothesized that alterations in circadian rhythm, suppression of melatonin secretion, and modulation of serotonin may be important contributory factors.^9,10^ Light is the strongest synchronizer of circadian rhythms, with the circadian effects of light acting through the eyes via the retinohypothalamic tract, a direct neural pathway from the retina to the suprachiasmatic nucleus, which is recognized as the central biological clock in the brain.^11^ More recently, it was shown that melanopsin, a photopigment located in retinal ganglion cells, modulates the circadian effects of light.^12^ Melanopsin is particularly sensitive to wavelengths of light in the blue color range (i.e., 450 to 480 nm),^12^ and low-intensity blue light can shift circadian rhythms as effectively as higher-intensity white light.^13^ In addition, melanopsin plays an important role in suppressing melatonin production, as well as improving alertness and neurobehavioral performance, which may mediate some of the antidepressant effects of light.^14,15^ There is also evidence that blue light may promote affective arousal and modulate emotional brain responses, notably in areas involved in depression such as the amygdala, hippocampus, and hypothalamus.^16^ These recent findings suggest specific wavelength hypotheses for light therapy for depression, including (1) blue light at low intensity may be efficacious, which could have advantages such as fewer side effects and shorter treatment time than higher intensity white light, and (2) enriching high-intensity white light with blue wavelengths (we will refer to this as blue-enhanced white light throughout the text) may be more effective than standard white light.

To our knowledge, there are no quantitative syntheses looking specifically at the effects of blue-light therapy on patients with seasonal and non-seasonal MDD. Previous meta-analyses of light therapy focused on non-seasonal MDD and did not distinguish between specific wavelengths of light.^4,5^ Our objective is to systematically review the literature on randomized controlled trials (RCTs) using blue light for depression and to examine the efficacy and safety of blue light for depressive disorders. We will compare blue light to inactive and active control conditions. We will also conduct sensitivity analyses for studies using low-intensity blue light (defined as below 1,000 lux) and for studies that included only participants with SAD.

## Methods

### Literature Search and Study Selection

The systematic review was registered with PROSPERO (www.crd.york.ac.uk/prospero/, CRD#42021239374) and conducted following Preferred Reporting Items for Systematic Reviews and Meta-Analyses (PRISMA) guidelines, but a protocol was not published. The systematic search included studies up to January 17, 2022, and was conducted using the following databases: Web of Science, EMBASE (OVID), Medline (OVID), PsycInfo, and Clinicaltrials.gov. The search strategy contained the following terms: (blue OR blue-enhanced OR blue-enriched OR narrow-band OR wavelengths OR spectrum) AND (phototherap* OR “light therapy” OR “light treatment”) AND (depress* OR “seasonal affective disorder” OR bipolar). Medical subject heading (MeSH) terms were included when available. To look for additional studies that may have not been captured by the original database search, we performed backwards reference chaining by searching through bibliographies of relevant articles.

Studies were included if: (1) they were published RCTs; (2) the active intervention was low-intensity blue light or blue-enhanced white light; (3) participants met diagnostic criteria (e.g., DSM-IV, DSM-5, ICD-10) for a major depressive episode, seasonal or non-seasonal; and (4) a clinician-rated measure of depressive symptomatology was used (e.g., Hamilton Depression Rating Scale [HDRS],^17^ Montgomery-Åsberg Depression Rating Scale [MADRS]^18^). Studies were excluded if: (1) the participants had other comorbid conditions as a primary diagnosis; and (2) the active intervention included a combination of light therapy with another treatment (e.g., sleep deprivation) and the comparison condition did not include the other treatment. Abstracts, case reports, case series, and review articles were also excluded.

Two independent reviewers (AD, VWL) screened titles and abstracts of articles retrieved by the search for inclusion. Potentially eligible articles were further reviewed by reading the full text. Any initial disagreements between the reviewers were resolved by joint review, discussion, and consensus or through consultation with an independent third reviewer (RWL).

### Data Extraction

Two independent reviewers (AD, VWL) extracted the data using a data extraction form designed for the study. Any disagreement was resolved by consensus or through consultation with a third reviewer (RWL). Study authors were contacted if eligible data were not reported in the paper. The following data were extracted: participant demographics (mean age, sex, and primary diagnosis), study characteristics (design, duration, inclusion/exclusion criteria, sample size, dropouts, and reasons for dropout), details of the active intervention, and comparison conditions, outcomes measures and scores at baseline and endpoint, response/remission rates, and adverse events.

### Risk of Bias and Quality Assessment

We used version 2 of the Cochrane risk-of-bias tool for RCTs^19^ to assess bias in the following categories: randomization process, deviations from interventions, missing outcome data, measurement of the outcome, selection of reported outcomes, and overall bias.

### Statistical Analysis

The primary outcome was the difference in endpoint scores on the Structured Interview Guide for the Hamilton Depression Rating Scale - Seasonal Affective Disorder (SIGH-SAD)^20^ or the Structured Interview Guide for the Hamilton Depression Rating Scale with Atypical Depression Supplement (SIGH-ADS)^21^ between the active and comparison conditions. The SIGH-SAD is a 29-item clinical interview comprising the 21-item HDRS with 8 additional items for atypical depressive symptoms. The SIGH-ADS is a 25-item clinical interview that includes the 17-item HDRS with the same 8 items for atypical symptoms. Secondary outcomes included: (1) clinical response (≥ 50% improvement from baseline to end of treatment score on a clinician-rated depression rating scale), and (2) clinical remission rates (endpoint score in the remission range). We also examined tolerability using acceptability (all-cause discontinuations) and dropouts due to adverse events.

All outcomes were analyzed with the intent-to-treat samples (ITT) if available. Since all the studies (except Danilenko 2019) used either the SIGH-SAD or SIGH-ADS as the main clinician-rated depression rating scale, the primary outcome was analyzed using mean differences (MD) as a measure of effect size. If endpoint scores were not available, change scores (endpoint minus baseline scores) were used. The secondary outcomes were analyzed using odds ratios (ORs). Outcome data were extracted at the end of treatment for each study unless otherwise specified. As per Cochrane recommendations,^22^ to guard against the inflation of effect size for studies with more than one intervention arm, either the results of all active treatment arms were pooled as one intervention or the control group size was divided by the number of active intervention arms. For crossover studies, we included only data for the first arm of the crossover.^22^

For our primary analyses, we compared blue-light conditions, which include both low-intensity blue and blue-enhanced white light, to inactive conditions (e.g., low-intensity red light) and separately to active conditions (e.g., high-intensity white light). In addition, we conducted sensitivity analyses of (1) studies using low-intensity blue light to test the hypotheses that low-intensity blue light has efficacy compared to inactive conditions or may be equal or superior to white light, and (2) studies including only SAD participants.

Since we anticipated heterogeneity in study methodologies, such as variations in the type of depression (seasonal MDD vs. non-seasonal MDD), study duration, and type of control condition, we used a random-effects model. Statistical heterogeneity was assessed using Q chi-square statistics and *I*^2^; an *I*^2^ of 50% to 70% suggests moderate heterogeneity and 75% to 100%, high heterogeneity.^23^ Publication bias was determined with: (1) funnel plots of outcomes plotted against their standard error, (2) Rosenthal's fail-safe *N* (the number of unidentified negative studies that would need to exist to change the result),^24^ and (3) Egger's regression intercept (a statistical test to examine asymmetry in the funnel plot).^25^ The Trim and Fill procedure was used to impute missing studies if publication bias was suggested.^26^ The quantitative meta-analysis was done using Comprehensive Meta-Analysis Version 2.0 software (Biostat, USA).

## Results

### Study Selection

**Figure 1.**
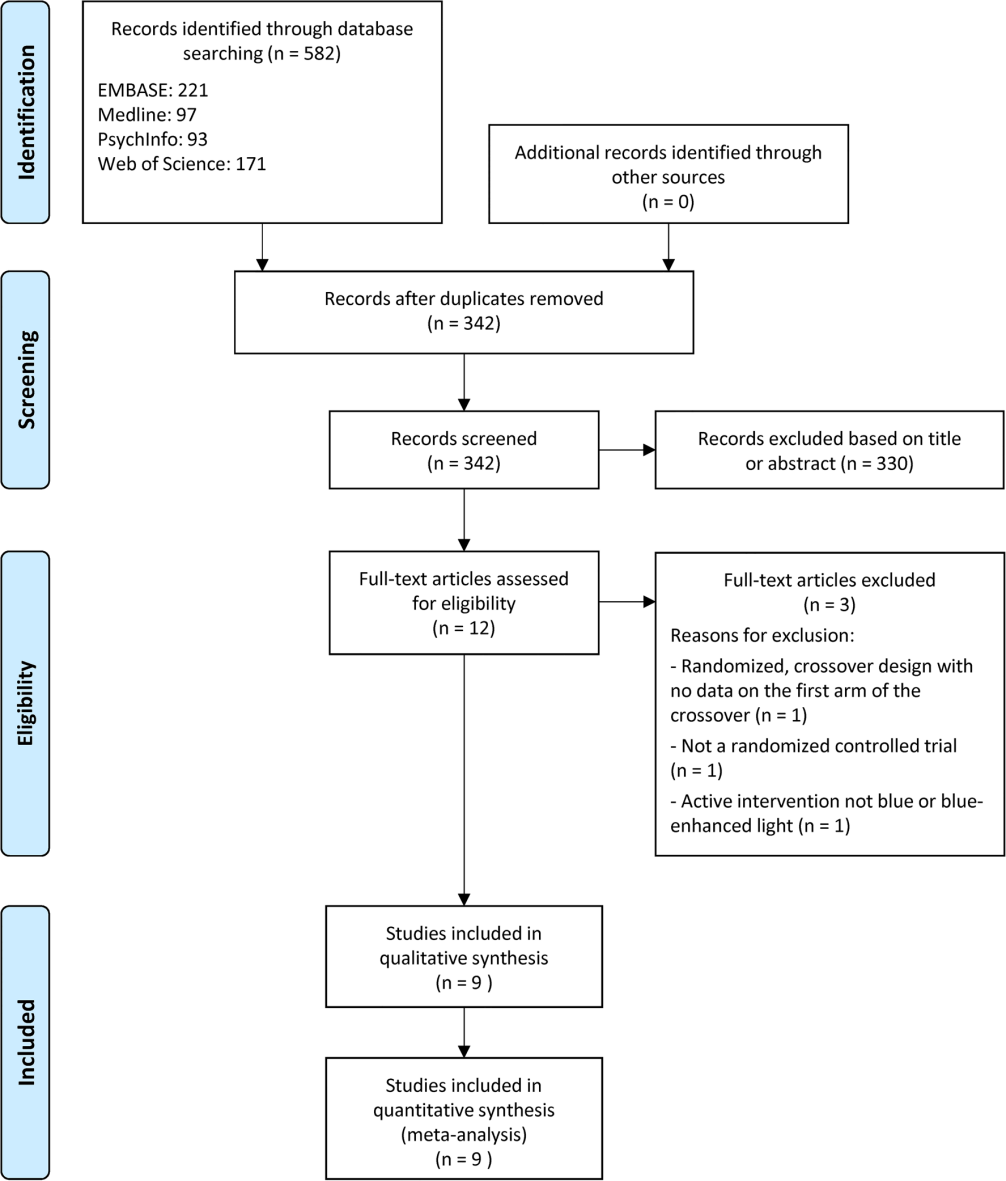
PRISMA flow diagram showing the number of database search results and article selection.

Figure 1 shows the PRISMA flow diagram for the literature search and study selection. The systematic search resulted in 582 articles. Following the removal of duplicates, 342 articles were screened via title or abstract review, after which 12 articles were assessed for eligibility via full-text review. Three articles were further excluded because the data on the first arm of the crossover was not available,^27^ the design was not randomized,^28^ or the active intervention was not low-intensity blue or blue-enhanced white light,^29^ resulting in a total of nine studies for the meta-analysis.^30–38^

### Study Characteristics

Table 1 shows the main characteristics of the nine included RCTs (*n* = 347 participants). Seven studies included participants with a primary diagnosis of SAD (MDD with seasonal pattern), and two studies (Danilenko 2019, Lieverse 2011) recruited patients with non-seasonal MDD; all the participants were diagnosed according to the DSM-IV, DSM-IV-TR, or DSM-5 criteria. One study (Anderson 2009) included three patients with bipolar II disorder, but the authors did not analyze the data separately for the bipolar subgroup. All the studies involved middle-aged participants, except for Lieverse et al., which included older patients. Most studies had small sample sizes, ranging from 18 to 84 participants.

**Table 1.** Characteristics of Included Studies.

Study/design	Diagnosis	Sample size (*n*)	Mean age (years ± SD)	Sex: male/female (*n*/*n*)	Treatment condition; wavelength; intensity	Control condition; wavelength; intensity	Daily light exposure	Study treatment duration	Outcome measure
Anderson et al., 2009/comparison	MDD with seasonal pattern (DSM-IV)	Tx: *n* = 9; Control: *n* = 9	Tx: 49.4 ± 6.5; Control: 48.7 ± 12.2	Tx: 3/6 Control: 3/6	Blue LED device; 464 nm; 98 lux	White LED device enriched in short-wavelengths; peak 460 nm; 711 lux	45 min	21 days	SIGH-ADS
Anderson et al., 2016/efficacy	MDD with seasonal pattern (DSM-IV-TR)	Tx: *n* = 18; Control: *n* = 17^a^	Tx: 49.9 ± 11.2; Control: 38.9 ± 11.4	Tx: 6/12 Control: 3/14	Blue-appearing LED device; 465 nm; 149.2 ± 12.1 lux	Orange-appearing LED device; peak 595–612 nm; 119.6 ± 21.3 lux	30 min	42 days	SIGH-ADS
Danilenko et al., 2019/comparison	MDD, recurrent or single episode or dysthymia, with melancholic or atypical features (DSM-5)	Tx: *n* = 19; Control: *n* = 16	Tx: 50.9 ± 10.8; Control: 49.7 ± 12.3	Tx: 10/9 Control: 10/6	Blue-enriched white light LED; 450 nm; 600–2,800 lux	Orange lens glasses blocking wavelengths < 540 nm and reducing light intensity by 70%	60 min or 240 min	6 days	HDRS-17
Glickman et al., 2006/efficacy	MDD with seasonal pattern (DSM-IV)	Tx: *n* = 13; Control: *n* = 13	Overall: 44.38 ± 2.62	Tx: 2/9^b^ Control: 3/10	Blue LED; 468 nm; 398 lux	Red LED; peak 654 nm; 23 lux	45 min	21 days	SIGH-SAD
Gordijn et al., 2012/comparison	MDD with seasonal pattern (DSM-IV-TR)	Tx (BLUE30): *n* = 18; Tx (BLUE20): *n* = 17; Control: *n* = 17	Tx (BLUE30): 37.9 ± 2.6; Tx (BLUE20): 39.3 ± 2.4; Control: 39.2 ± 3.4	Tx (BLUE30): 4/14 Tx (BLUE20): 5/12 Control: 3/14	Blue-enriched white light fluorescent box; 440 and 550 nm; 9,000–10,000 lux	White light fluorescent box; peak 550 and 620 nm; 9,000–10,000 lux	30 or 20 min	10 days	SIGH-SAD
Lieverse et al., 2011/efficacy	Non-seasonal MDD (DSM-IV)	Tx: *n* = 40; Control: *n* = 44^c^	Tx: 69.67 ± 8.5; Control: 69.00 ± 6.6	Tx: 14/28 Control: 17/30	Pale blue light fluorescent box; 440 and 550 nm; 7,500 lux	Red light fluorescent box; peak 620 nm; 50 lux	60 min	21 days	SIGH-SAD
Meesters et al., 2011/comparison	MDD with seasonal pattern (DSM-IV-TR)	Tx: *n* = 11; Control: *n* = 11	Tx: 41.7 ± 13.1; Control: 39.9 ± 12.7	Tx: 2/9 Control: 3/8	Blue-enriched white light fluorescent box; 440 and 550 nm; 750 lux	Standard white light fluorescent box; peak 620 nm; 10,000 lux	30 min	10 days	SIGH-SAD
Meesters et al., 2018/comparison	MDD with seasonal pattern (DSM-IV-TR)	Tx: *n* = 24; Control: *n* = 21	Tx: women = 37.06 ± 13.36/men 46.67 ± 14.46; Control: women = 35.56 ± 13.15/men = 39.8 ± 11.41	Tx: 6/18 Control: 5/16	Blue-enriched white light fluorescent box; 470 nm; 100 lux	White fluorescent light box; unspecified wavelength; 10,000 lux	30 min	5 days	SIGH-SAD
Strong et al., 2009/efficacy	MDD with seasonal pattern (DSM-IV)	Tx: *n* = 15; Control: *n* = 15	Tx: 51.1 ± 12.3; Control: 39.5 ± 9.9	Tx: 5/10 Control: 2/13	Blue LED; 470 nm; 176 lux	Red LED; peak 650 nm; 201 lux	45 min	21 days	SIGH-SAD

^a^ Although 35 participants were randomized, data were available only for 29/35.

^b^ Sex distribution is only reported for completers.

^c^ Although 89 participants were randomized, 84/89 were included in the statistical analysis.

DSM = Diagnostic and Statistical Manual of Mental Disorders; HDRS = Hamilton Depression Rating Scale; LED = light emitting diode; MDD = Major Depressive Disorder; min = minutes; nm = nanometers; SD = standard deviation; SIGH-ADS = Structured Interview Guide for the Hamilton Depression Rating Scale - Atypical Depression Supplement; SIGH-SAD = Structured Interview Guide for the Hamilton Depression Rating Scale - Seasonal Affective Disorder Version; Tx = Treatment condition.

Eight of the included RCTs involved daily light exposure without sleep deprivation for at least 5 days; one (Danilenko 2019) used a 6-day protocol consisting of partial sleep deprivation alternating with morning light treatment, but both treatment groups received sleep deprivation. The light parameters for both the active and comparison conditions varied across studies. For the active condition, six studies (Anderson 2009, Anderson 2016, Glickman 2006, Meesters 2011, Meesters 2018, Strong 2009) used low-intensity blue light, while two studies (Gordjin 2012, Lieverse 2011) used blue-enhanced white light. The blue light intensities ranged from 98 to 10,000 lux. The blue light wavelength remained consistent across studies, ranging from 450 to 470 nm. For the comparison conditions, four studies (Anderson 2009, Gordjin 2012, Meesters 2011, Meesters 2018) used standard white light (intensity ranging from 711 to 10,000 lux), three (Glickman 2006, Lieverse 2011, Strong 2009) used dim red light (intensity ranging from 23 to 201 lux), one (Anderson 2016) used an orange-appearing medium-wavelength light (average intensity of 120 lux) and one (Danilenko 2019) used orange-appearing glasses blocking wavelengths below 540 nm. For all the studies except Danilenko et al. and Gordjin et al., the daily light exposure was either 30, 45, or 60 min per day. The Gordjin et al. study had three treatment conditions: (1) 30 min standard light therapy, (2) 30 min blue-enhanced white light therapy, and (3) 20 min blue-enhanced white light therapy. The study treatment durations varied from 5 to 42 days.

### Risk of Bias Assessment

**Figure 2.**
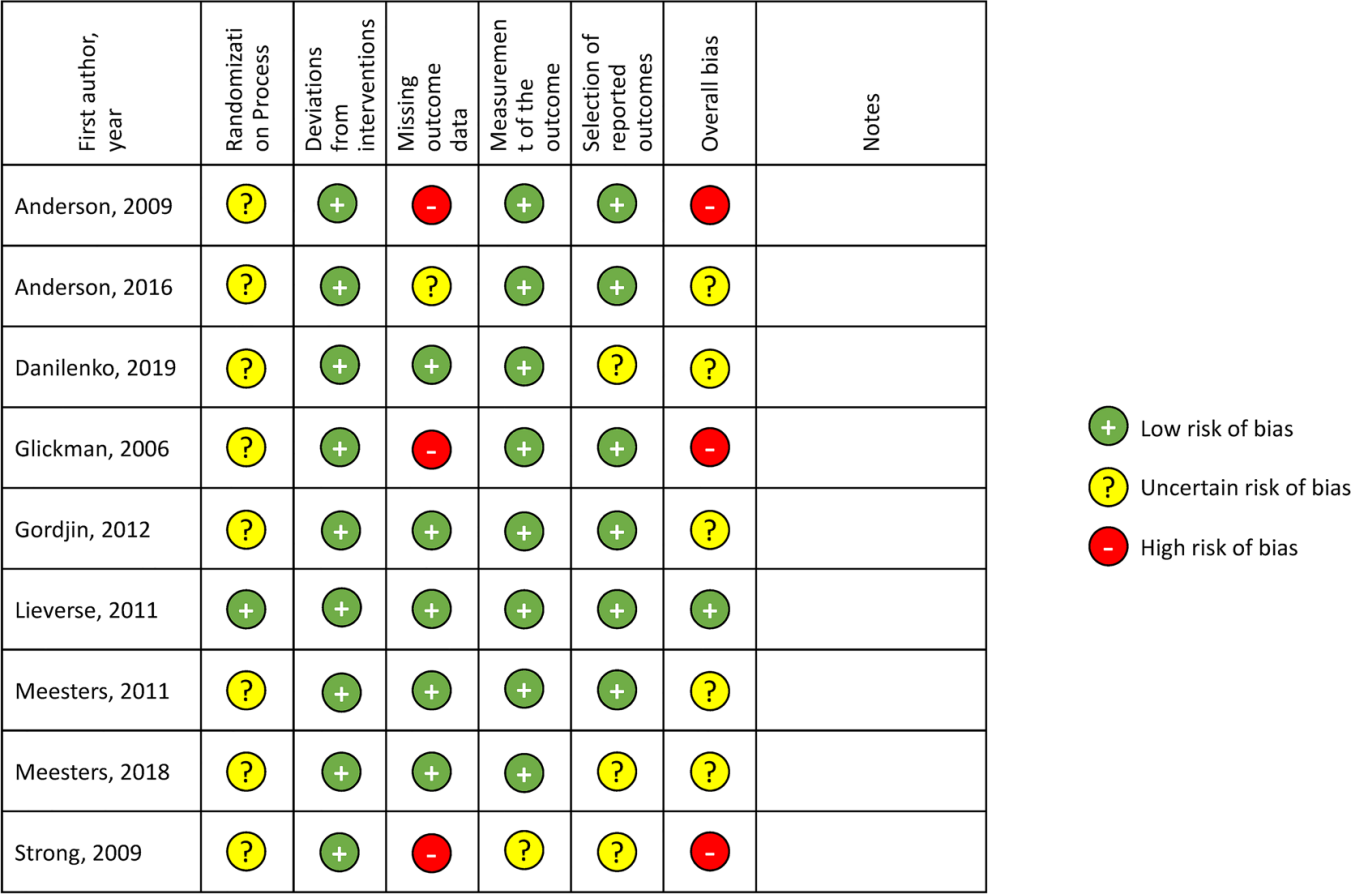
Risk of bias assessment for included studies.

Figure 2 shows the summary of the risk of bias assessment from the Cochrane risk-of-bias tool. Except for Lieverse et al., all the studies showed an uncertain or high risk of bias in at least one methodological category of risk. Only one study (Lieverse 2011) described the allocation concealment process and had a low risk of bias for the randomization process. None of the studies had a high risk of bias for deviations from interventions, measurement of the outcome, or selection of reported outcomes. For selective reporting, pre-specified analysis plans were not available for three studies (Danilenko 2019, Meesters 2018, Strong 2009), and as a result, they were assessed to have an uncertain risk of bias. Four studies (Anderson 2009, Anderson 2016, Glickman 2006, Strong 2009) had incomplete information on dropouts. A potential risk of bias for several studies (Anderson 2009, Anderson 2016, Gordjin 2012, Meesters 2018, Strong 2009) was that light device manufacturers funded the study.

### Meta-Analysis

The primary outcome was the difference in endpoint scores on the SIGH-SAD or SIGH-ADS between blue-light therapy and comparison conditions. Six studies reported endpoint scores; Strong et al. reported change scores and Glickman et al. reported a between-group mean difference. We excluded Danilenko et al. from the primary outcome analysis because it was the only study that did not use either the SIGH-SAD or SIGH-ADS. Since the control conditions included inactive and active conditions, we reported them separately. Studies comparing blue light to an inactive condition were considered efficacy studies, while studies comparing blue light to an active condition were considered comparison studies.

### Efficacy Studies

**Figure 3.**
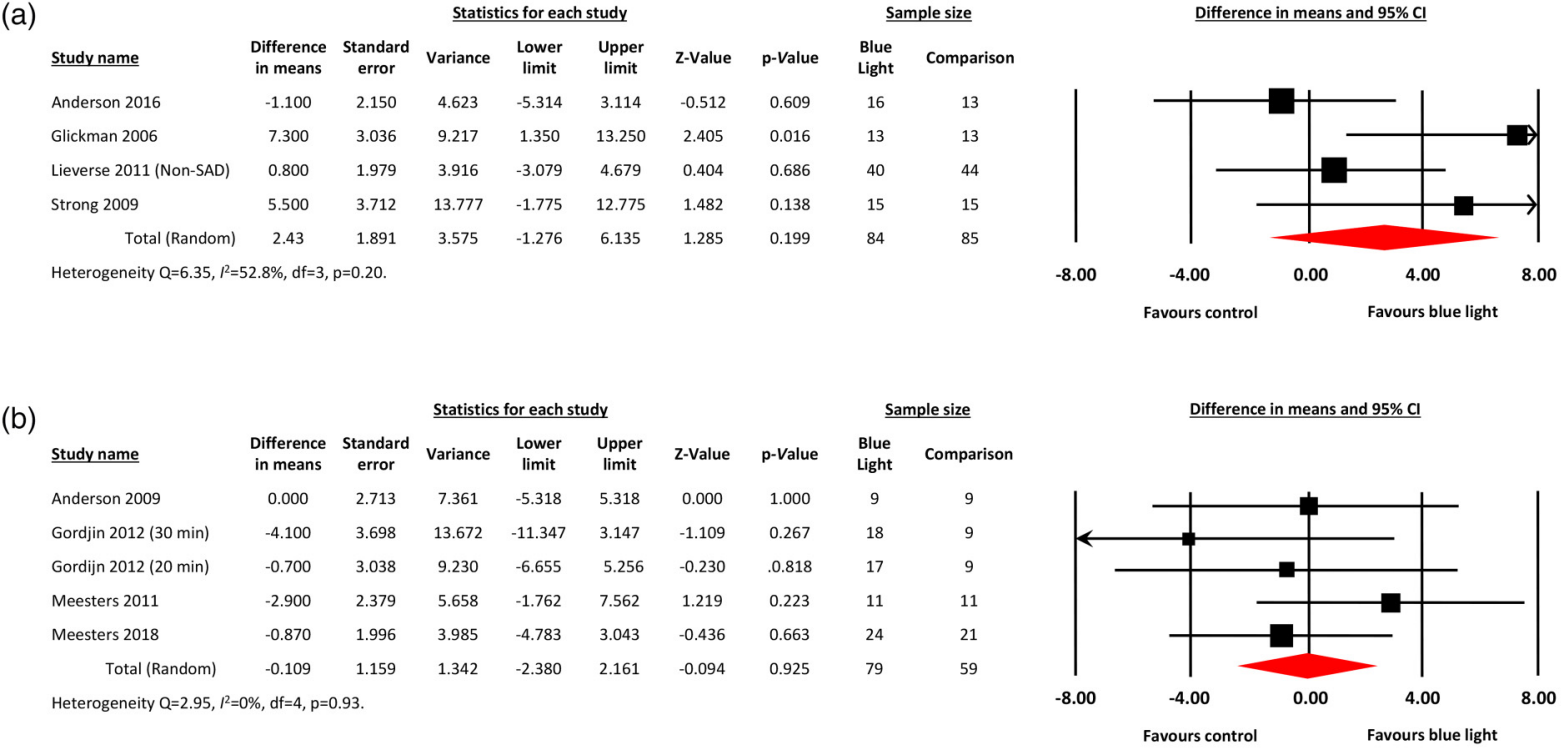
Forest plots displaying meta-analyses of (A) mean differences in endpoint scores for blue-light therapy versus inactive control conditions; (B) mean differences in endpoint scores for blue-light therapy versus active control conditions.

Figure 3 shows the forest plots for blue light and control conditions. For efficacy studies, there was no difference between blue light and inactive conditions in clinician-rated depressive symptoms (MD = 2.43; 95% confidence interval [CI], −1.28 to 6.14, *P* = 0.20; 4 studies, total n = 169 participants) (Figure 3A). There was moderate heterogeneity between the studies, with *Q* statistic of 6.35 and *I*^2^ of 52.8% (degrees of freedom [df] = 3; *P* = 0.10). The funnel plot of standard errors by effect size estimates was broadly symmetrical, the fail-safe *N* was 0 since the outcome was nonsignificant, and the Egger's intercept was 4.38 (two-tailed *P* = 0.17), suggesting a low probability of publication bias.

For response rates, comparison to inactive conditions produced an OR of 1.93 in favor of blue-light therapy that was not statistically significant (95% CI, 0.96 to 3.88; *P* = 0.07; 3 studies, *n* = 149) (Supplementary Materials). There was no heterogeneity between the studies, with a *Q* statistic of 1.38 and *I*^2^ of 0% (df = 2; *P* = 0.50). The unadjusted response rates for blue light and inactive conditions were 56% and 41%, respectively. For remission rates, comparison to inactive conditions revealed an OR of 1.21 (95% CI, 0.43 to 3.43; *P* = 0.72; 2 studies, *n* = 61) (Supplementary Figure 1B). There was no heterogeneity between the studies, with a *Q* statistic of 0.57 and *I*^2^ of 0% (df = 1; *P* = 0.45). The unadjusted remission rates were 55% for blue light and 50% for inactive conditions.

### Comparison Studies

For comparison studies, there was no difference between blue light and active conditions in the endpoint SIGH-SAD or SIGH-ADS scores (MD = −0.11; 95% CI, −2.38 to 2.16, *P* = 0.93; 4 studies, *n* = 138) (Figure 3B). There was no heterogeneity between the studies (*Q* statistic = 2.95; *I*^2^ = 0%; df = 4; *P* = 0.57), and a low probability of publication bias (symmetrical funnel plot of standard errors; fail-safe *N* = 0; Egger's intercept = −1.68 with two-tailed *P* = 0.45).

There were also no significant differences between blue light and active conditions in response and remission rates (Supplementary Figure 1C and D). For response rates, the OR was 1.17 (95% CI, 0.36 to 3.82; *P* = 0.80; 4 studies, *n* = 154) with moderate heterogeneity between the studies (*Q* statistic = 6.07; *I*^2^ *=* 50.6%; df = 3; *P* = 0.11). The unadjusted response rates for blue light and active conditions were 69% and 66%, respectively. For remission rates, the OR was 0.72 (95% CI, 0.35 to 1.49; *P* = 0.38; 4 studies, *n* = 150) with no heterogeneity between the studies (*Q* statistic = 2.30; *I*^2^ = 0%; df = 3; *P* = 0.51). The unadjusted remission rates for blue light and active conditions were 54% and 60%, respectively.

### Sensitivity Analysis

In total, six studies used low-intensity blue light ranging from 98 to 750 lux. These studies included only participants with SAD. For efficacy studies in SAD, there was no difference between low-intensity blue light and inactive conditions (MD = 3.47; 95% CI, −2.23 to 9.17, *P* = 0.23; 3 studies, *n* = 85). There was moderate heterogeneity between the studies (*Q* statistic = 5.95; *I*^2^ *=* 66.4%; df = 2; *P* = 0.05). For comparison studies in SAD, low-intensity blue light performed similarly to active conditions (MD = 0.52; 95% CI, −2.09 to 3.13, *P* = 0.70; 3 studies, *n* = 85). There was no heterogeneity between the studies (*Q* statistic = 1.52; *I*^2^ = 0%; df = 2; *P* = 0.47).

### Acceptability and Adverse Events

Regarding acceptability (all-cause discontinuation rates), three studies did not specify in which group they occurred, so a meta-analysis was not possible; instead, we provide a qualitative review. Common reasons for all-cause discontinuation included a poor response to treatment (Anderson 2009, Anderson 2016, Glickman 2006), scheduling conflicts (Anderson 2009, Glickman 2006), inability to follow treatment schedule (Anderson 2009), medication switch (Lieverse 2011), worsening of depression (Lieverse 2011) and medical illness (Danilenko 2019, Meesters 2011, Meesters 2018).

Similarly, data on dropout rates due to adverse events were reported inconsistently across studies and some studies did not specify in which treatment group they occurred, hence a meta-analysis was not possible and instead we provide a qualitative review. Overall, both blue-light therapy and the comparison conditions were well tolerated. The most common side effect associated with blue-light therapy was headache. In total, three participants (two in the Anderson et al. 2016 and one in the Strong et al. 2009 studies) dropped out prematurely due to side effects related to the study device. In the Anderson et al. study, one patient in the blue light group dropped out after experiencing a migraine and a “hot spot on the eye,” while one patient in the white light group dropped out due a combination of headache, migraine, eye strain and the report of a white flashing light. The Strong et al. study did not specify why the patient dropped out. No switches to hypomania or mania were described, but four studies did not report on switch rates.

## Discussion

To our knowledge, this is the first systematic review and meta-analysis of RCTs of blue-light therapy for seasonal and non-seasonal MDD. Overall, we found mixed results for efficacy and comparison studies. The meta-analysis found no significant difference between blue-light therapy and comparison conditions (either active or inactive conditions) in reducing the primary outcome of depressive symptom scores as measured by the SIGH-SAD or SIGH-ADS. Hence, there is no current evidence supporting the *efficacy* of blue-light therapy since our primary and sensitivity analyses for studies with inactive conditions were both negative. On the other hand, the comparison studies revealed that blue light and active conditions performed similarly in both primary and sensitivity analyses. This may suggest that low-intensity blue light could be as *effective* as standard light therapy conditions; however, it must be pointed out that the comparison between blue-light therapy and active conditions was not powered for non-inferiority. Regarding safety and tolerability, although we were unable to provide a quantitative synthesis, the qualitative review suggested that blue-light therapy was generally well-tolerated by patients, with only mild side effects and no serious adverse events or hypomanic/manic switches reported.

These results should be interpreted with caution given the limitations of our meta-analyses. First, the included studies had variable heterogeneity and quality. For example, the light parameters for both active and inactive conditions, duration of daily light exposure, and duration of study treatment varied considerably across all the studies. Both seasonal and non-seasonal depression were included, although the sensitivity analyses with only SAD studies were no different than the primary analyses. One study (Anderson 2009) also included three patients with bipolar disorder. Regarding study quality, only one of the nine studies had an overall low risk of bias. Second, the included studies had small sample sizes that were likely underpowered, limiting their ability to detect a treatment effect. Third, since all the studies were of short treatment duration (with the longest duration only 6 weeks, in one study), the longer-term effects of blue-light therapy remain unstudied.

Given these limitations, there are several possible explanations for the lack of efficacy of blue-light therapy reported in our meta-analysis. First, the small number of included studies may have led to the non-rejection of a false null hypothesis (type II error). Second, the benefit of blue-light therapy may take longer to demonstrate than the short treatment duration of the included studies. For example, clinical trials have suggested that the separation of active light conditions from sham conditions may take four weeks or longer.^1^ Third, there was considerable heterogeneity in the type of light device used, ranging from larger light boxes to small, portable LED devices. Compared to standard fluorescent light boxes, these smaller devices may have greater variability in the positioning of the patient during use, leading to suboptimal light exposure to the eyes. Finally, although low-intensity blue light can suppress melatonin and shift human circadian rhythms,^39^ the circadian effects of light may not mediate its antidepressant effects. In SAD, the evidence to support a circadian hypothesis for the antidepressant mechanism of light therapy remains sparse,^40^ and alternate mechanisms have been hypothesized, including non-circadian effects of bright light on neurotransmitters such as serotonin and dopamine.^9,10,41^ The lack of difference between blue light and active conditions may be the result of placebo effects since the comparison studies did not include a placebo control condition to validate the efficacy of the active condition, or due to small sample sizes.

In summary, there is no current evidence for the efficacy of blue-light therapy in the treatment of seasonal and non-seasonal MDD, according to our meta-analysis. However, given our finding that blue light performed similarly to active conditions, better quality studies are needed to demonstrate the efficacy of blue light in depression. Future trials of blue-light therapy should be of longer duration, include larger sample sizes, and attempt to better standardize the parameters of light therapy. Further investigation is also necessary to determine the optimal dosing parameters (e.g., intensity, spectra, duration of daily exposure) of light therapy for both seasonal and non-seasonal depression.

## Supplemental Material

sj-docx-1-cpa-10.1177_07067437221097903 - Supplemental material for Blue-Light Therapy for Seasonal and Non-Seasonal Depression: A Systematic Review and Meta-Analysis of Randomized Controlled Trials Click here for additional data file. Supplemental material, sj-docx-1-cpa-10.1177_07067437221097903 for Blue-Light Therapy for Seasonal and Non-Seasonal Depression: A Systematic Review and Meta-Analysis of Randomized Controlled Trials by André Do, Victor W. Li, Samantha Huang and Erin E. Michalak, Edwin M. Tam, Trisha Chakrabarty, Lakshmi N. Yatham, Raymond W. Lam in The Canadian Journal of Psychiatry

sj-pptx-2-cpa-10.1177_07067437221097903 - Supplemental material for Blue-Light Therapy for Seasonal and Non-Seasonal Depression: A Systematic Review and Meta-Analysis of Randomized Controlled Trials Click here for additional data file. Supplemental material, sj-pptx-2-cpa-10.1177_07067437221097903 for Blue-Light Therapy for Seasonal and Non-Seasonal Depression: A Systematic Review and Meta-Analysis of Randomized Controlled Trials by André Do, Victor W. Li, Samantha Huang and Erin E. Michalak, Edwin M. Tam, Trisha Chakrabarty, Lakshmi N. Yatham, Raymond W. Lam in The Canadian Journal of Psychiatry
