# Supplementary material for: Blue-Light Therapy for Seasonal and Non-Seasonal Depression: A Systematic Review and Meta-Analysis of Randomized Controlled Trials
Source: Can J Psychiatry. 2022 May 6;67(10):745–54. doi: 10.1177/07067437221097903 (PMC9511000; doi:10.1177/07067437221097903)
Supplement: sj-pptx-2-cpa-10.1177_07067437221097903 - Supplemental material for Blue-Light Therapy for Seasonal and Non-Seasonal Depression: A Systematic Review and Meta-Analysis of Randomized Controlled Trials [file sj-pptx-2-cpa-10.1177_07067437221097903.pptx]

## Slide 1
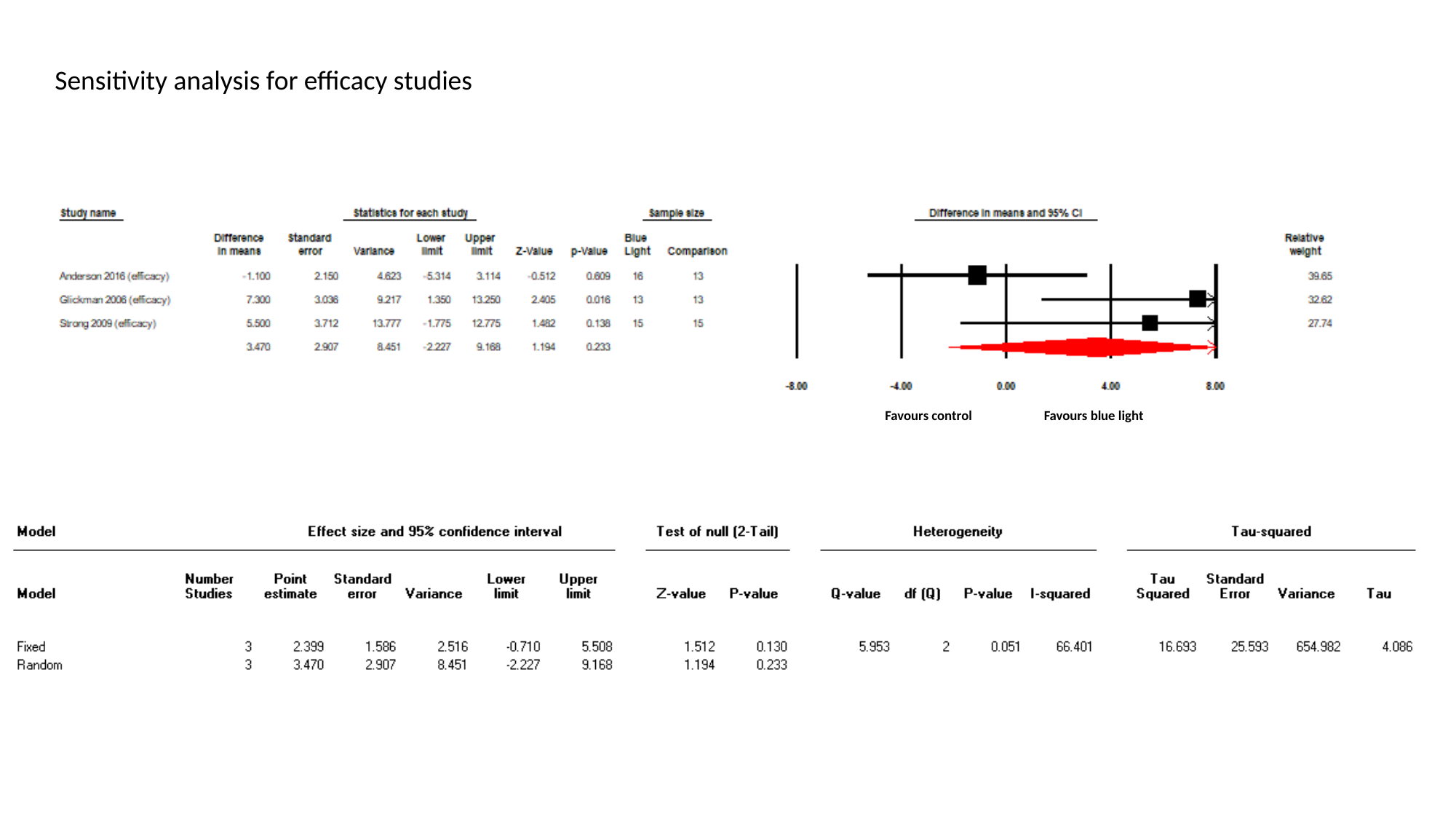

Sensitivity analysis for efficacy studies
Favours control
Favours blue light

## Slide 2
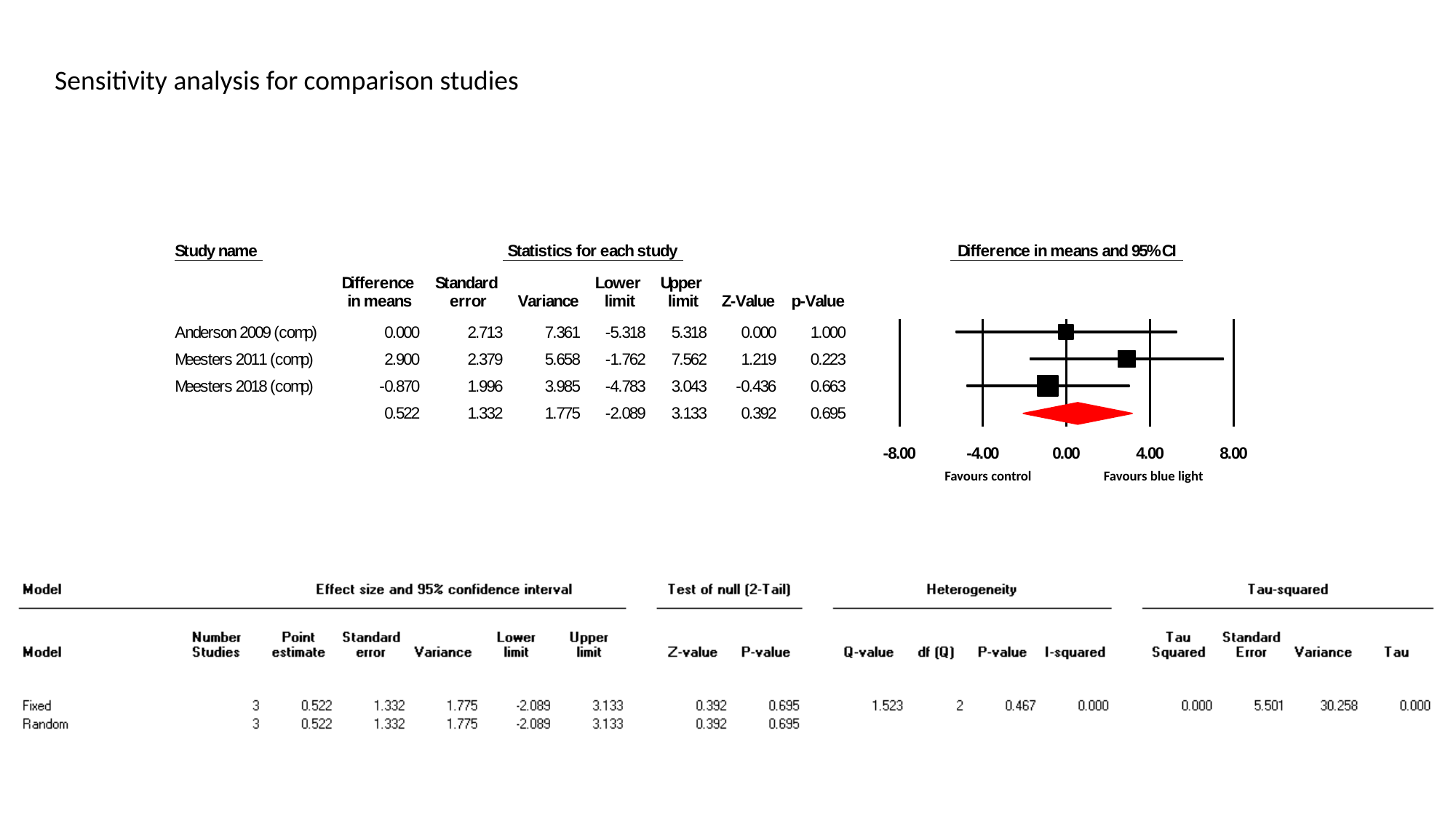

Sensitivity analysis for comparison studies
Favours control
Favours blue light
